# An Evaluation of the Chicago Public Schools Gender Inclusivity Professional Learning Community for K-12 Teachers

**DOI:** 10.1007/s11256-025-00802-3

**Published:** 2026-01-22

**Authors:** Elizabeth Jarpe-Ratner, Janet Kamiri-Ong, Laura Decker, Faven Habte, Kristen Belcher, D. Little, Booker Marshall

**Affiliations:** 1 University of Illinois Chicago, 1603 W. Taylor St., Chicago, IL 60613 USA; 2 Chicago Public Schools, Office of Teaching and Learning, Chicago, IL USA; 3 Chicago Public Schools, Office of Network Services, Chicago, IL USA; 4 Chicago Public Schools, Office of Student Health and Wellness, Chicago, IL USA; 5 The Potocsnak Family Division of Adolescent and Young Adult Medicine, Ann & Robert H. Lurie Children’s Hospital, Chicago, USA

**Keywords:** LGBTQIA+ students, Science teachers, Science curriculum, Inclusive curricula, School health and wellness, Professional learning communities

## Abstract

An increasing number of K-12 public school students report holding lesbian, gay, bisexual, transgender, queer or questioning, intersex, or asexual or aromantic (LGBTQIA+) identities. And, we know these students are at significantly higher risk of experiencing a variety of negative health outcomes, especially transgender and gender expansive students who are at greatest risk. That said, a strong evidence-base demonstrates that LGBTQIA+ students in schools with protective policies and inclusive curricula experience significantly improved outcomes. Some districts, like Chicago Public Schools (CPS), are implementing these evidence-based policies and programs to protect LGBTQIA+ students. Yet, there remain opportunities to grow and expand this work, including: (1) exploring models for professional development that best equip teachers with the skills needed to implement these programs and policies; and (2) identifying more ways that curricula, including science curricula, can be inclusive. With these opportunities in mind, CPS launched a Gender Inclusivity Professional Learning Community (GI PLC) in 2022 with 7 participants. This case study describes how the GI PLC functioned to support teacher learning and how teachers developed projects, including curricula, that they took back to their classrooms. In 2023, 10 interviews were conducted with PLC facilitators (N = 4), participants (N = 5), and a technical assistance provider (N = 1). Interviews were recorded, transcribed, and thematically analyzed using MaxQDA qualitative data analysis software. Key findings revealed that participants reported value in terms of learning and community-building. The aims and content of the projects that participants developed within the GI PLC were aligned with: understanding students’ identities at an individual level; developing inclusive curricula at a classroom level; and leading policy implementation at the school level. The GI PLC can be a model for other districts working to support LGBTQIA+ students through curricula and engaging in multi-level change approaches.

## Introduction

Students in K-12 school systems are increasingly sharing their identities as lesbian, gay, bisexual, transgender, queer or questioning, intersex, or asexual or aromantic, (LGBTQIA+). In 2015, 18.5% of Chicago Public Schools (CPS) high school students indicated their LGB identity on the Youth Risk Behavior Survey, while in 2023, 26.9% of high school students identified as LGB and an additional 2.2% identified as transgender. In the same year, 31.1% of middle school students identified as LGB and 1.9% of middle school students identified as transgender (CDC, 2003–2023). Unfortunately, LGBTQIA+ youth are more likely to have experienced bullying, violence, depression, days missed from school, and an array of negative health outcomes (CDC, 2003–2023). Despite these statistics, there is strong evidence demonstrating that LGBTQIA+ students can thrive in schools where their identities are affirmed (Ioverno et al., 2016; Jarpe-Ratner et al., 2023; Kosciw et al., 2022; Mayo, 2018; Mayo & Blackburn, 2019; Truong et al., 2021). Students in schools that protect against bullying on the basis of sexual orientation and gender identity (Russell et al., 2021); have LGBTQIA+ inclusive curriculum (Russell et al., 2021); display signs and symbols of support; have Gender and Sexuality Alliances (GSA) clubs (DeMartino & Weiser, 2025; Russell et al., 2021), and provide support for gender transition, are more likely to have experienced school connectivity, report a sense of belonging, and become connected to a trusted adult at school (Kosciw et al., 2022). Many schools, including CPS, are adopting these protective strategies, including increasing support for Gender and Sexuality Alliance clubs, ensuring protections against bullying based on gender or sexual orientation, and ensuring social studies curricula include historical LGBTQ+ figures. There remains, however, an opportunity to engage more deeply in how science curricula are addressing the diversity of sex and gender.

CPS’s efforts to support LGBTQIA+ students are led by its Office of Student Health and Wellness (OSHW). OSHW is nationally recognized for their work to create, implement, and evaluate a yearly asynchronous professional development (PD) requirement for all staff in the district (Jarpe-Ratner & Little, 2024). Further, their comprehensive, LGBTQIA+-inclusive sexual health education policy has been cited as a model for other districts (Moore, 2022). Districts play a profound role in creating policy and adopting evidence-based strategies and CPS is a leader in its sustained work in this area over the past decade (Jarpe-Ratner et al., 2022).

Despite all this, implementing these practices across such a large district remains extremely challenging. OSHW leaders recognized two opportunities for continued growth in SY 22–23. First, was an opportunity to explore additional professional development models. While quantitative data showed significant learning gains and attitudinal shifts following the mandated asynchronous PD for all staff members (Jarpe-Ratner et al., 2023), qualitative data collected from school staff routinely demonstrated a desire for more engaged training opportunities and spaces to discuss strategies and lessons learned (Jarpe-Ratner et al., 2022). Second, while policies requiring inclusive curricula for sex education and social studies had been in place in the district, OSHW leaders identified an opportunity to explore models for inclusive curricula in other content areas, particularly in science, leading to the creation of the Gender Inclusivity Professional Learning Community (GI PLC) in 2022. The GI PLC focused on creating spaces for teachers to explore how to create safe and supportive environments in schools through inclusive curricula. This paper documents the creation of the GI PLC and its initial evaluation to explore the GI PLC’s influence on teachers’ learning and their creation of materials for classrooms and schools, including but not limited to gender inclusive science curricula. This evaluation was intended to inform ongoing professional development and curricular efforts in the district and may have important lessons learned for other districts engaging in similar efforts.

## Literature Review: Supporting LGBTQIA+ Students in Schools and the Role of Inclusive Curricula

There are two areas of literature that inform the rationale for and the development of the GI PLC in CPS. These are (1) evidence for professional development strategies supporting LGBTQIA+ students in schools and (2) literature on inclusive curricula, including science curricula.

### Supporting LGBTQIA+ Students in Schools Through PD: The Current Evidence

Professional development for educators on how to support LGBTQIA+ students in schools is an evidence-based strategy through which teachers and staff are trained to recognize and respond to bias, use inclusive language, and support LGBTQIA+ students, effectively enhancing the overall school climate and ensuring that inclusion is embedded in everyday practice (Jarpe-Ratner et al., 2023; McDermott et al., 2024; Richard, 2015). Schools that have implemented LGBTQIA+ inclusivity training for teachers have seen reduced disciplinary incidents, including lower rates of suspensions, assaults, and endangering behaviors (McQuillan et al., 2023; Wright et al., 2025). Training teachers can be an essential step in ensuring that evidence-based strategies, such as anti-bullying policies and gender support policies, are actually implemented in schools and experienced by LGBTQIA+ students.

Different models of PD have been evaluated. Evidence suggests that sustained professional development programs—those lasting several months and involving structured sessions, mentoring, and community engagement—are more effective in fostering lasting changes in educator attitudes and practices toward LGBTQIA+ inclusion (McQuillan et al., 2023; Zapletal et al., 2024). To date, there are few or no evaluations specifically of Professional Learning Communities (PLCs) in the United States used to instill skills to support LGBTQIA+ students in schools. One in Canada, led by Guerrero and colleagues (2017) documented the development of a PLC, “Engaging All Students,” for K-12 educators in Toronto. It aimed to support teachers in the implementation of “culturally relevant and responsive” teaching practices for marginalized students in the district. PLC facilitators found that educators’ understanding of race and culture were often simplified to refer to one’s ethnicity, race, or ancestry, and “overlooked how student experiences related to their gender, gender identity, and/or self-identification with the LGBTQ communities” (Guerrero et al., 2017, p. 6). As such, facilitators engaged in activities with educators to expand this understanding of the intersection of race, ethnicity, and culture with students’ gender identity and sexual orientation (Guerrero et al., 2017). That said, there have been calls for such an approach. In their text, *LGBTQ + Studies in Education: Theoretical Interventions in Curriculum and Pedagogy*, Mizzi & Rodriguez (2025) discuss how collaborative reflection, a core element of PLCs, can and should be used as a mechanism to foster skills among educators to support LGBTQIA+ students in schools. This guide advocates for collaborative learning sessions where educators explore the experiences and needs of LGBTQIA+ youth, reflect on their own biases, and develop actionable strategies to create safer school environments (Mizzi & Rodriguez, 2025).

### Inclusive Curricula: Impact on Students and Opportunities in Science Curricula

Evidence reveals that inclusive curricula also play a vital role in supporting LGBTQIA+ students. When LGBTQIA+ perspectives, histories, and contributions are integrated into classroom content, LGBTQIA+ students report feeling seen, validated, and included. This sense of belonging is associated with improved mental health, greater academic engagement, and stronger educational outcomes. Inclusive curricula also help reduce bias and bullying by educating all students about diversity and equity. Evidence-based practices include incorporating LGBTQIA+ authors and historical figures into literature and history lessons, teaching about diverse family structures, and using gender-neutral language in classroom materials. Recent reviews show that inclusive curricula foster resilience, reduce depression, and promote acceptance among students (Dang, 2024; Schlief et al., 2023).

Despite positive outcomes associated with LGBTQIA+ inclusive curricula, inclusive curricular materials tend to be used infrequently, especially in science (Snapp, Burdge, et al., 2015; Snapp, McGuire, et al., 2015). A mere 2.0% of LGBTQIA+ students reported experiencing inclusive curriculum in their science classes in 2021–2022 (Kosciw et al., 2022). Wright and Delgado (2023) emphasize the pervasiveness of heteronormativity in science curricula. Science textbooks purporting to be an authority on biology often default to simplistic explanations of gender, sex and sexuality that are informed by the heteronormative default (Wright & Delgado, 2023). Even when sexual and gender diversity are represented in science curricula, it is often framed as strange, surprising, or a curiosity (Rende Mendoza & Johnson, 2024).

Nevertheless, there are gender diverse educators who have created frameworks for inclusive science curriculum. Long, Steller, and Suh (2021) propose five attributes in adapting science curricula to be inclusive: authenticity, continuity, affirmation, anti-oppression, and student agency. They stress that being accurate and representative of biological diversity across species necessarily invites gender inclusivity (Long et al., 2021). As Rende Mendoza & Johnson (2024, p. 964) point out in their analysis of Long et al. (2021), the framework for Gender-Inclusive Biology emphasizes that “encouraging students to ask questions, develop their own language, and giving them a protected space to voice concerns can create safer conditions for them to engage in material that may be deeply personal.” Given the demonstrated need and emerging frameworks for inclusive science education, there is opportunity to adapt existing curricula and interest from educators to do so.

This examination of the literature reveals that not only did CPS have an opportunity to explore PD models and inclusive science curricula, but there are opportunities in the wider field of LGBTQIA+ support in schools for such models and practices.

## Chicago Public Schools: A National Leader with Opportunities in PD and Science

As noted above, CPS is a national leader in supporting LGBTQIA+ students. As the 4th largest district in the nation, serving over 300,000 students in over 600 schools, approximately 80% of whom are students of color and over 70% are classified as “economically disadvantaged” (Chicago Public Schools, 2025), CPS’s work in this area is transferable to many other large, diverse districts. For over a decade, OSHW, with financial support from the Centers for Disease Control and Prevention’s Division of Adolescent and School Health (CDC-DASH), supports these efforts (Centers for Disease Control and Prevention Division of Adolescent and School Health, 2018). OSHW has developed a required professional development (PD) for all staff covering basic LGBTQIA+ content and best practices for supporting transgender and gender nonconforming students; invested in the creation and sustainment of GSAs across the district; and has provided coaching and technical assistance to the more than 600 schools in the district on supporting LGBTQIA+ students and staff members’ rights.

Additionally, state legislative efforts in Illinois have expanded curriculum requirements in specific content areas to provide more inclusive instructional experiences. The Keeping Youth Safe and Healthy Act of 2021 requires that sexual health education curriculum is “medically accurate, developmentally and age-appropriate” and includes instruction that is also “culturally appropriate, inclusive, and trauma-informed.” Public Act 101–0227, also known as the Inclusive Curriculum Law, which took effect in 2020, addressed social science curricula by mandating that Illinois and US history “shall include a study of the roles and contributions of lesbian, gay, bisexual, and transgender people in the history of this country” ("Illinois Inclusive Curriculum Law,"). To date, no state legislation or CPS policies have directly addressed gender inclusivity in science curricula.

### Covering Sex and Gender in Science Education in CPS

The CPS high school science curriculum was updated in alignment with the Next Generation Science Standards (NGSS) in 2014 (Illinois State Board of Education, 2016). The curriculum provides resources for biology, chemistry, and physics, and encourages teachers to modify and/or supplement instructional materials according to their professional judgment. Neither the CPS curriculum nor NGSS address sex or gender directly and instead approach the topics through the normative study of sexual reproduction. Both employ scientific phenomena as a tool to build student understanding which can often encourage a simplified, “depoliticized approach to teaching… that reinforce[s] binary perspectives” (Mattheis et al., 2022, p. 15). These gaps, along with the ability to adapt curricular materials, create an opportunity to provide a more inclusive learning experience that teaches the complexity of sex and gender in the natural world and disrupts prevalent and harmful misconceptions.

### PLCs in CPS

Over the past 5–10 years, CPS has allocated many resources and funding streams to create and sustain Professional Learning Communities (PLCs) to support teacher professional development on a variety of topics. These spaces focus on building teacher curriculum knowledge, facilitation, and differentiation while also creating space for teachers to expand their own knowledge and ideas around equity within the learning environment (Mundy et al., 2015; Woodland, 2016). PLCs in CPS aim to: (1) afford teachers a space to share, and inquire into their teaching practice; (2) foster collaboration among teachers; (3) center learning around evidence-based practice, student data, and student-reported experiences; (4) leverage the expertise and diversity of the group; and (5) center ongoing improvement (Mundy et al., 2015; Prediger et al., 2019; Woodland, 2016).

As noted above in the literature review, there have been calls to create spaces for ongoing critical reflection to better equip teachers with the skills needed to support LGBTQIA+ students in schools. That, coupled with the fact that PLCs were becoming more common in the district, paved the way for the creation of the GI PLC.

## Theoretical Foundation

The choice to create a PLC not only aligned with calls from the literature and CPS’s prioritization of PLCs, it also aligned with adult learning theory as described below. The PD approach aligned well with the district’s existing multi-level approach to supporting LGBTQIA+ students.

### Theoretical Foundation for PLC Approach: Adult Learning and Pedagogy

Continuous forms of professional development, such as PLCs are considered a form of adult learning (Stoll et al., 2006; Woodland, 2016; Zepeda et al., 2014). The GI PLC, incorporates components of adult learning theory, such as “action, experiential, self-directed, and project-based learning,” (Zepeda et al., 2014, p. 299).

Effective forms of adult learning should be built on “ownership, appropriateness, structure, collaboration, internalization, reflection, and motivation” (Zepeda et al., 2014, p. 300). This PLC was informed more specifically by the 7 principles of adult learning proposed by Elena Aguilar: (1) Adults must feel safe to learn; (2) Adults come to learning experiences with histories; (3) Adults need to know why we have to learn something; (4) Adults want agency in our learning; (5) Adults need practice to internalize learning; (6) Adults have a problem centered orientation to learning; and (7) Adults want to learn (Aguilar & Cohen, 2022).

### SEM: A Framework for Supporting LGBTQIA+ Students in CPS

To effectively support LGBTQIA+ students and staff, CPS uses the Social Ecological Model (SEM) to guide its work, ensuring that strategies span multiple ecological levels of the school environment (Bronfenbrenner, 1977; McQuillan et al., 2024; Veldhuis, 2022). Multilevel interventions such as these go beyond individual behavior change strategies and increase the likelihood of sustainable systems change (Golden & Earp, 2012). In CPS, the policies to support LGBTQIA+ students (e.g. Guidance: Gender & Sexuality Protections (Chicago Public Schools, 2024)) function at the policy level, Genders and Sexualities Alliances (GSAs) function at the organizational/school level, and the required professional development (PD) for all staff in the district (Jarpe-Ratner et al., 2023) functions at the individual and interpersonal levels. Inclusive curricula also function at the individual level, helping affirm students in their identities. They also function the interpersonal level fostering dialogue and collective learning in an inclusive space, as well as at the school and district levels as curricula are adopted and implemented at those levels. PD plays a crucial role in supporting all levels of implementation as it is intended to drive the implementation of policy- and organizational-level interventions through individual skills, behavior, and actions to implement and reinforce programs and policy.

## The Gender Inclusivity PLC

Originally, the PLC’s intended focus was on gender inclusivity in science curricula and classroom environments. Science teachers were recruited for the monthly meetings using a flyer and direct emails. Based on initial feedback from interested teachers, the focus on curriculum development evolved to align with the district’s goals to support LGBTQIA+ students more broadly and ensure inclusivity of both gender identity and sexual orientation. In order to continue to center gender identity, the name of the PLC continues to be referred to as the Gender Inclusivity (GI) PLC.

In alignment with principles of adult pedagogy described above, the team wanted to ensure that this learning was a cooperative effort and space where all members of the PLC community could share their ideas around building quality classroom communities for all students, think through solutions for more inclusive curriculum, and create safe spaces for this learning to take place. While the PLC had overarching goals, participants were given agency to pursue projects and topics that felt meaningful and relevant to their work and school contexts with the intention of bringing the product back for implementation in their classrooms, departments, and/or schools, as appropriate. Biology educators from the local zoo, including both of whom identify as transgender and/or nonbinary, participated in the PLC and offered Technical Assistance (TA) to participants.

Initial meetings focused on developing norms and learning about the importance of gender inclusivity, the policies and protections of LGBTQIA+ students in CPS, and activities to assess the current curricula, including a curriculum audit activity. At the end of the fall 2022, participants began to identify specific projects they would work on based on what they had learned, discussed, reflected upon, and critiqued during these first few sessions. The remaining monthly meetings were focused on participants having work time to develop their projects. PLC facilitators and TA providers would circulate and serve as coaches or thought partners to help participants develop their projects. Teachers were compensated for their time attending meetings as well as independent work time on their projects between meetings. Finally, during the last meeting held in May, participants presented their projects to one another.

## Aims and Purpose of Evaluation

As described above, OSHW leaders recognized a need to create PD spaces that allowed for group dialogue, reflection and action and there was a need to support the creation of gender inclusive curricula in science. Therefore, the GI PLC was formed as a collaborative effort among CPS staff from OSHW, Office of Network Services (ONS) that support PD, and Office of Teaching and Learning (OTL) that support curricula, as an avenue to explore how to address these needs. In an effort to maximize learning about this new approach and inform changes to the PLC as a model in the future, CPS collaborated with the University of Illinois Chicago (UIC) School of Public Health (SPH) to conduct a developmental evaluation (Patton, 2010). The evaluation aimed to explore how a PLC facilitates learning among teachers about how to better support their LGBTQIA+ students. Specifically, the evaluation explored the following questions: (a) What did teachers learn as a result of their participation in the PLC?; (b) What products were created for teachers to take back to classrooms and schools, including but not limited to gender inclusive science curricula?; (c) How did the key characteristics of PLCs influence teacher learning and experiences?; and (d) What are recommendations for future PLCs related to supporting LGBTQIA+ students? This qualitative case study was intended to inform ongoing professional development and curricular efforts in the district and may have important lessons learned for other districts engaging in similar efforts.

## Evaluation Design and Methods

This developmental evaluation (Patton, 2010) is just one component of larger evaluation collaboration between CPS OSHW and UIC SPH supported by a cooperative agreement with the CDC DASH to support efforts in public schools to create safe and supportive environments for LGBTQIA+ and all youth (Centers for Disease Control and Prevention Division of Adolescent and School Health, 2018). The goal of developmental evaluation is to evaluate a new approach in real-time, in collaboration with program staff, and use data to inform ongoing program improvements and adaptation (Patton, 2010). This approach was selected in part because, as we noted in the literature review, there are few examples of using a PLC for skill development related to supporting LGBTQIA+ students. Developmental evaluation is appropriate for such novel, emergent approaches (Patton, 2010). Additionally, program staff wanted the data to immediately make program changes to be implemented the following year. This evaluation was reviewed and determined non-research by the UIC Institutional Review Board and was reviewed and approved by the CPS Research Review Board, the oversight body that reviews all district data collection, whether deemed research or not.

### Interview Sampling and Participation

This study sought to understand the experiences among facilitators of the GI PLC as well as those participating in it and supporting it through TA. Therefore, all facilitators (those staff members from CPS OSHW, ONS, and OTL who created, launched, and facilitated the PLC), participants (teachers who participated in the PLC), and TA providers (local experts in biology education) were invited to participate. Evaluators attended a PLC meeting to discuss the opportunity to participate in an interview and emails were sent as a follow-up for scheduling. All interviews were held virtually via Zoom between April and May of 2023. Each session lasted approximately 1 hour, was digitally recorded, and was transcribed. All PLC facilitators and participants were invited to participate. All four PLC facilitators were interviewed, five (out of a total of nine PLC participants) completed an interview, and one (of two) technical assistance providers (biology educators from the local zoo) were interviewed for a total of ten interviews.

### Interview Guide Development

An interview guide included questions about how the PLC was initiated and/or how the interviewee got involved (e.g. “Why did you get involved in the PLC?”; “What were your expectations going into the process?”), their understanding of its purpose (e.g. “Was there a clear goal for the GI PLC, and if so, what was it?”), and their overall perceptions and experiences of the PLC (e.g. “What are you taking away from this experience?”; “What was new to you?”; “Have you learned any new knowledge or skills and if so, how have you used them, if at all?”). In alignment with our employment of a PLC as a method for adult learning we wanted to inquire about key characteristics of PLCs known to be associated with critical reflection and adult learning as identified by Stoll and colleagues in a systematic literature review (Stoll et al., 2006). Stoll and colleagues identified six key characteristics of PLCs and we included questions and prompts on the interview guide related to each one including: (1) clear goal and purpose; (2) established norms and values; (3) presence of an overarching organizational structure; (4) activities and resources aligned with the goals; (5) space for collaboration and relationship building; and 6) critical thinking and reflection. OSHW, ONS, and OTL stakeholders reviewed the guide for appropriateness of language, flow, and sequencing (Krueger & Casey, 2009).

### Analytic Approach

Two coauthors (E.J.R., K.B.) used thematic analysis that incorporated a hybrid (both inductive and deductive) approach including *a priori* codes from the literature about PLCs as well as emergent codes, using constant comparative methods (Corbin & Strauss, 2015; Padgett, 2012). Examples of a priori codes drawn from the PLC literature mentioned above included, “purpose and goals,” “norms and values,” “activities and resources,” "collaboration and relationships,” “organization and structure,” and “critical thinking and reflection.” Examples of emergent codes mostly related to outcomes, including “school level outcomes,” and “district level outcomes.”

First cycle coding involved identification of initial themes by the coders who compared and discussed their coding application on 2 transcripts. Initial agreement rates were calculated using MaxQDA’s intercoder “percent agreement” function, calculating the basic proportions of matching coded segments to the total number of coded segments. Areas of disagreement were reviewed by both analysts and discussed, and coding definitions were changed to make them more distinct and clearly applicable to both coders. Once the codes were refined and applied to the second transcript, an 80% agreement rate was exceeded and then the remaining transcripts were coded by a single coder. The same two study authors met regularly to review coding applications and discuss emerging themes through a peer debriefing process (Lincoln & Guba, 1985). Upon review of coded text segments, second cycle coding was conducted, and analytic memos were written to identify key themes and the relationships among them (Corbin & Strauss, 2015; Kuckartz & Rädiker, 2023). These preliminary analyses were shared with the rest of the authorship team and further refined for clarity and brevity.

### Positionality of Authors

The members of our authorship team hold a diverse array of educational and disciplinary backgrounds, demographic identities and lived experiences, current roles and associated professional expertise as well as prior experiences as classroom teachers. Four co-authors are current district office employees, one is a former district office employee, and two are members of the evaluation team (one faculty member and one doctoral student). Three members have graduate training in education while four are former K-12 teachers including two as former high school biology teachers. Four members have graduate training in public health and four have previously held community-based public health positions. We drew heavily on our expertise in public health in framing the PLC as part of a multilevel public health approach as well as our collective background in education and pedagogy which informed our approach to adult learning, student learning, and curriculum. Three authors hold queer identities, including one who identifies as a transmasculine, non-binary person. Four authors identify as people of color and three identify as first-generation Americans. While the authorship team's collective demographic identities do not exactly mirror that of the district's students or teachers, the team engaged in systematic reflection and dialogue to explore both their collective strengths their identities and experiences bring as well as the biases and the ways they could limit their understanding.

## Findings

The findings from the interviews with the twelve participants are presented below. The interviewees included four PLC facilitators, 5 PLC participants, and 1 technical assistance provider from a local community-based organization. The findings are organized according to the evaluation questions guiding the study, including: (a) What did teachers learn as a result of their participation in the PLC?; (b) What products were created for teachers to take back to classrooms and schools, including but not limited to gender inclusive science curricula?; (c) How did the key characteristics of PLCs influence teacher learning and experiences?; and (d) What are recommendations for future PLCs related to supporting LGBTQIA+ students and how have these been incorporated? The first question is answered descriptively, themes from the thematic analysis are reported for the second and third questions, and the fourth question is also answered descriptively.

### What Did Teachers Learn?

The participants described several outcomes they themselves had experienced as a result of their participation in the PLC. First, it reinforced the importance of addressing gender diversity and LGBTQIA+ inclusion in the curriculum and classroom practices. One stated, “...we need to bring this home to the buildings in a different way… this [is] really about people's existence and their lives and their community” (Interviewee #5). Many participants felt empowered to make concrete changes, such as revising language and examples used in lessons to be more inclusive. One participant noted, “so that I can use this and I can share it because it is important to, to talk about and to, and to have opportunities to, you know, teach science through a lens that is not often taught through” (Interviewee #8).

Additionally, the collaborative nature of the PLC allowed participants to learn from each other's experiences and perspectives, which was particularly meaningful for those who did not previously have much exposure to these topics. Participants appreciated having a dedicated space and time to work on projects related to gender and sexuality, which they felt was lacking in their regular school contexts, “…just being able to start writing a unit that I think would be interesting for students and connecting it to things that I find important and make a difference for my students” (Interviewee #2).

Overall, the PLC fostered a sense of community and collective purpose around this work, which participants saw as crucial given the challenges and resistance they may face elsewhere. One participant shared, “…so I think just knowing that there is support, that in fact there are many best practices that exist. There are many people with this expertise…. that we are here and, and we are in community with each other now” (Interviewee #10). The process encouraged self-reflection on how to be better advocates and allies for LGBTQIA+ students. Participants left the PLC feeling more equipped and motivated to continue integrating gender diversity into their teaching practices.

### What Products Were Created?

#### Projects Extended Beyond Curricula

As noted above, and as is common of a PLC approach, facilitators aimed to create a space where teachers could create projects that aligned with their own goals, interests, and their perceptions of needs. Despite the initial focus on gender-inclusive curricula, through the discourse and reflection that took place during the first few meetings, participants identified larger needs that extended beyond the need for gender-inclusive curricula. For example, one participant noted,

...when I started doing some digging… then the conversation became, you know, like, we do the Safe Schools Training [mandated asynchronous webinar] and we have students that are probably not even aware of their rights. And we've overheard these conversations [referring to staff refusing to use students’ affirmed names and pronouns]. And so what's then an effective way to work us towards this like, sunny ideal that we had, you know, come up with… Which was like a, you know, a student facilitated, you know, workshop type thing about like student rights and stuff like that [to do with all the teachers in their school]. (Interviewee #4)

Similarly, another participant wanted to create more relationships with their students, “But you need to take time one-on-one to, to discuss, to [have] open discussion with, with each student. It takes time. But if you take time to do it, you will learn every student…” (Interviewee #3). Therefore, a variety of projects emerged that extended beyond curricula.

### Projects Aligned with the Social-Ecological Model (SEM)

Upon data collection and initial analysis, analysts realized that the projects aligned with the levels of the SEM, as described above in the district’s theoretical approach to its support for LGBTQIA+ students as described above. Projects are described in Table 1.

**Table 1:** Participant projects

Project	Project description	Project goals
Curricula Review	• Teachers reviewed the Skyline biology curriculum for gender inclusivity and made suggestions on areas where activities and language are not gender inclusive	• Review CPS science curricular materials and give recommendations for revision based on gender inclusivity
Lesson on sex and gender roles	• This biology unit utilizes natural occurrences of animals that change gender or exhibit gender fluidity within their respective populations for the survival of the species	• Design a unit that is intentionally gender inclusive • Use science content to show examples within nature where gender transition and fluidity happen outside of humans. • Create a space for conversations around gender, LGBTQIA+ issues, and inclusivity
Lesson on physics and house music history	• This physics education project incorporated the LGBTQIA+ and BIPOC history of house music in Chicago. The goal was to create a more inclusive curriculum that resonates with students from these communities, to increase belonging	• Design a physics unit that is intentionally gender inclusive. • Include local history to increase the relevance of physics content to students
Staff professional development	• This project focused on staff professional development for the staff and teachers at this participant's specific school. The professional development session introduced participants to district-level data with a focus on LGBTQIA+ student experiences and health outcomes and shared best practices for creating safe, affirming, and gender-inclusive spaces for students	• Understand the defining characteristics of the students in the district. • Identify the first steps to ensure students of all identities feel safe and welcome in their classrooms. • Share resources for students at the school
Student survey	• This project focused on the importance of affirming student identity in the classroom. The participant created a written survey tool as well as a teacher-student interview protocol to support beginning of the school year conversations around identity	• Create a beginning-of-the-year survey for teachers to use to get to know students leading to an increased ability for teachers to create curriculum and classroom spaces that are more responsive to the needs and interests of their students

Two curricular projects functioned at the individual learning and classroom level. One was a biology lesson intended to encourage students to critique assumptions of binary sex and gender among animal species. The teacher designed a biology unit utilizing natural occurrences of animals that change gender or exhibit gender fluidity within their respective populations for the survival of the species. The teacher noted, “So looking at different gender roles within other organisms and seeing that they're not all the same at all, but they all play a role in survival.” (Interviewee #8). This teacher emphasized the importance of the TA provided by facilitators and TA providers, especially those who identify as transgender, expressing a desire to ensure the lesson was not, “focusing too hard on gender roles” and thereby reinforcing them.

The other curricular project was a physics project focused on the physics of music, tying in the history of LGBTQIA+-centered house music in Chicago. This physics education project incorporated the LGBTQIA+ and BIPOC history of house music in Chicago, connecting the historical and cultural aspects of music with the underlying electronics and physics of the instruments. The teacher noted, “So like looking at the actual electronics in it and then that's how it, that's how I want to connect it to the physics” (Interviewee #2). This teacher’s goal was to create a more inclusive curriculum that resonates with students from the communities where House music came from. The teacher also emphasized the ways that House music has created inclusive spaces for Black, Latino, and LGBTQIA+ populations in Chicago and elsewhere. The aim was to increase belonging, understanding, and appreciation, in addition to physics knowledge.

Another teacher created a student survey project that analysts determined was aligned with the interpersonal level. This project was intended to be used by teachers as a means to get to know their students better on a personal level and invite them to share elements of their identity with teachers, thereby fostering deeper relationships and understanding between teachers and students. According to Interviewee #3, the objective is to help teachers understand their students better, especially those who might be quiet or hesitant to share personal information, thereby promoting a more inclusive and successful learning environment. They emphasized that by allowing students to share they can better connect with students and foster a more inclusive learning environment, on a variety of identities, needs, interests, and strengths in addition to gender identity or sexual orientation.

Finally, aligned with the school level, two other participants created a professional development project intended to be used among staff at one school to facilitate conversations about how LGBTQIA+-inclusive policies can be embraced within schools. The professional development session introduced participants to district-level data with a focus on LGBTQIA+ student experiences and health outcomes and shared best practices for creating safe, affirming, and gender-inclusive spaces for students. Their goal was to collaborate with students in their school’s GSA (Genders and Sexualities Alliance club for LGBTQIA+ students and their allies) to co-facilitate the workshop.

These projects reflected an emphasis on student identity within the classroom and fostering ongoing professional development for educators to ensure that district-level policies intended to be inclusive of LGBTQIA+ students are understood by school staff and adhered to at the school level. Participants expressed their passion for their own projects and their enthusiasm for them as potential models for replication across the district once fully developed and piloted.

#### How Did the Key Characteristics of PLCs Influence Teacher Learning and Experiences?

As noted in the introduction, according to the PLC literature, PLCs are characterized by six elements: purpose and goals, norms and values, organizational structure, activities and resources, collaboration and relationship-building, and critical thinking and reflection (Stoll et al., 2006). Participants were asked about their experiences with the PLC vis a vis all six elements. Four themes were identified in the data as related to these six components. These are reported in Table 2 and are described in greater detail below.

**Table 2:** Themes and illustrative quotations aligned with key characteristics of PLCs

Themes	Illustrative quotations
1. PLC allowed for iteration and evolution of purpose over the course of the year	“… [We] talked about as it was going to be looking at the curriculum, trying to make that more gender inclusive… in the original conversations… there were all sorts [of ideas]... It's like, well, this is a problem, but this is also a problem and this is a problem, and all these problems are [interrelated]. And so in all that conversation, like there was really good conversation about all the places we could help improve the inclusion.” –Interviewee #4 “…When we first started, I wasn't really sure, like I kind of knew the overarching purpose, but one of the things that ultimately I liked most about it was that the group was able to pick what we thought were priorities and kind of work on, you know, a passion project… Some people were working on curriculum changes, other people were looking at making surveys, doing PDs. And so I kind of liked that it was a little nebulous at first. Like, okay, here's what we wanna work on in terms of themes, but what does this look like and what work is gonna be important for you in your building… and maybe other buildings if it, you know, moves beyond that.” --Interviewee #5
2. Values and norms allowed for learning and critical reflection	“I think that there was definitely space for collaboration towards the beginning... and I think that it was very clear how that stuff was laid out, you know, within the structure of the PLC... how that collaboration was built into the day. And it was so much like, it felt like, I don't know, I think with [facilitators] it was like, this is a, this is an activity we're gonna do at the beginning. Everyone know each other. And it just felt, it felt like very like, welcoming, collaborative." --Interviewee #1 “I think the debate around intention versus impact was really interesting… it was like the first time I really had heard it debated in like, this type of a setting… So I think that came up then later for me in other groups that I'm in where, you know, people said, you know, assume good intent. Then, you know, we talked, I was able to talk to those groups about like, well, I was in this other, you know, PLC where we talked about intent versus impact and, you know, maybe that's something we should be considering. So that was really helpful and I think the process would be really helpful for teachers as they're like, working on these topics with their students.”--Interviewee #6
3. Activities and resources facilitated critical reflection and creation of participants own projects	“I think some of the resources were really valuable… for me because… I felt like that's what I needed. So when those resources were provided to me, I was like, okay, cool. Like, this is new ways to teach this, these are different terms to use instead of using ‘this,’ use ‘this.’ …Hearing some of the other participants say, ‘Oh, I don't ever say female, male. I say parent one, parent two.” …So hearing what other people had to say as well. I liked the idea of the curriculum audit and us kind of delving through this curriculum and saying, what could you use here instead? Where are you gonna see potential issues of like the gender issue and so I did like the curriculum audit as well.” –Interviewee #8
4. Relationships and networking were valuable, yet participants craved more interaction and feedback on their individual projects	“What was nice about having those folks [from the zoo] is they were obviously very competent in this area, and they also do science education for youth in the context of their roles. So they're coming at it from a different angle… So they're, they're accustomed to teaching students about science in ways that are expanding people's understanding, uh, understanding of, of gender and other things. So I thought that that was super valuable. And then with, you know, [facilitator] and [facilitator] also are experts in education, science, education… it's nice to have people that are like working in the field every day… on different things that are bringing a different perspective that's outside of just K-12 education because… It's just good to get different perspectives.” –Interviewee #10

### PLC Allowed for Iteration and Evolution of Purpose

As reported above, the group evolved from an initial focus on curricula to more broadly focus on creating a safe and inclusive environment for LGBTQIA+ students, which included professional development for teachers and creating relationships with students. One noted, “I liked that, that we had all of these avenues and everybody kind of went into different lanes depending on where they, they were. And I think that was, I think that was good” (Interviewee #5). Participants also reported that with this evolving focus, the structure of the PLC evolved accordingly. While early sessions focused on building community, establishing norms, and providing foundational knowledge on gender inclusivity, participants realized there was more to address than just curriculum, “because it's kind of such a huge thing as we started to like, brainstorm, which again, we have the space to do that. Like, okay, what does this need to be?” (Interviewee #5). Therefore, the meetings became more open-ended, allowing participants to work independently on their own projects. This flexible structure was appreciated, as it gave participants the space to explore and develop their ideas.

#### Values and Norms Allowed for Learning and Critical Reflection

All participants were asked about whether the group had a shared set of norms and in what ways those norms or values functioned to support the group. Most participants shared the perceptions that the group had solid norms and boundaries from the first meeting, which allowed for open discussion and learning. Many specifically talked about learning about the difference between intent and impact (see Table 2). Additionally, participants described the importance of differentiating a growth mindset, noting: “Like, take this from a place of growth… not everybody in here is… you know, versed in using correct language. So like, let's keep in mind that this is a place for, you know, growth… everybody is here for the right reasons… they're here because they want to do better” (Interviewee #8).

Some noted that the small size of the group was a factor in how well the norms were established and maintained. One participant who missed the first meeting noted that there was an implicit shared set of norms and values based on respectful conversations and community building activities. The importance of creating a safe space for vulnerability and growth was emphasized by participants. The possibility of adding new members in the next year was discussed, and the idea of starting with group norms and values was suggested.

#### Activities and Resources Facilitated Critical Reflection and Project Development

In accordance with how the PLC was originally designed, interviewees reported that the PLC activities and resources focused primarily on curriculum development and integration of inclusive content, with an emphasis on biology and science topics. Participants and facilitators described a curriculum audit exercise that they found particularly valuable. This activity consisted of reviewing an existing biology lesson that loosely touched on the sex of different species and asked them to critically examine the lesson to identify opportunities to remove gendered language and incorporate more diverse examples. One interviewee noted, “I liked the idea of the curriculum audit and us kind of delving through this curriculum and saying, what could you use here instead? Where are you gonna see potential issues of like the gender issue…” (Interviewee #8). The interviewees also discussed how they had engaged in open-ended discussions that support critical reflection and generated many useful ideas and insights. One noted, “I personally like the time to mull it over, noodle it around before. Yeah. Cause when you jump in, that's when you're just like powering through and it doesn't always end with the best result (Interviewee #4). Participants appreciated the opportunity to share resources, particularly those related to gender identity and inclusivity, which they found more broadly applicable beyond the specific science content.

#### Relationships and Networking were Valuable, Yet Participants Craved More

Interviewees reported that the PLC facilitated meaningful connections and collaboration among participants, though the level of engagement varied over time. In the early sessions, there were structured activities and opportunities for participants to learn from each other and build relationships. One interviewee shared, “it was light. It was, they were fun and it wasn't like, uh, I don't know, it didn't seem very forced, you know?” (Interviewee #4). Participants noted that the involvement of the TA providers was seen as valuable and provided diverse perspectives. The TA provider interviewed, agreed, noting “So I definitely think that like, that blend and that through that conversation, it feels like it was a really meaningful connection, I feel like, for all of us in that moment (Interviewee #1).

However, interviewees noted that as people focused more on their individual projects, there was less collective sharing and dialogue. Participants appreciated the dedicated time and space to work on their projects, and many made new connections they wouldn't have otherwise. That said, both participants and facilitators stated they thought more could have been done to sustain the collaborative dynamic. Participants suggested incorporating more structured check-ins, presentations, and feedback mechanisms to maintain the collaborative spirit throughout. Overall, the PLC provided a welcoming and collaborative environment, though more consistent structure and feedback mechanisms could have further enhanced the learning experience for participants.

### What are Recommendations for the Future of this PLC?

Again, in alignment with developmental evaluation, interviewees made several recommendations for how to improve the PLC, many of which were incorporated into the PLC the next year. The recommendations made by participants and the ways these were addressed and incorporated the following year are shown in Table 3. There were four primary recommendations and associated changes to the PLC. First, in Year 2 there were more frequent meetings, some were conducted virtually, and the meeting dates were set at the beginning in response to participant feedback. Secondly, more time was allotted for participants to share progress on their projects and receive peer feedback through a modified Constructivist Tuning Protocol (Center for Leadership and Educational Equity, 2024). Thirdly, rather than allotting so much time to independent work as was done in the PLC’s first year, in the second year there was more time for collaboration. Time was allotted for participants to raise and discuss problems or concerns they were encountering related to the PLC themes and share ideas across projects. Finally, a resource folder was created so participants and facilitators could collect, share, and retrieve all the resources shared during the PLC meetings, and found by participants in their own research and project development.

**Table 3:** Recommendations and improvements

Recommendations made by participants in year 1	Improvements made in year 2 in response to year 1 recommendations
Meeting structure and frequency: Participants desired more frequent meetings, a mix of in-person and virtual sessions, and to set all meeting dates at the beginning to improve attendance	• PLC facilitators set all PLC session dates at the start of the school year and incorporated several virtual meetings to help decrease the burden of travel for meetings that were focused on workshopping projects together
Feedback: Participants wanted more time for discussion and feedback on their projects	• PLC facilitators incorporated a modified Constructivist Tuning Protocol multiple times throughout the PLC to give more structure to peer feedback sessions
Collaboration: Participants desired more time to mix up the groups and share ideas across projects	• PLC facilitators allotted time for participants to raise and discuss problems or concerns they were encountering at the school level related to inclusivity or project implementation and seek support from other PLC participants in addition to time to work on independent projects
Resource sharing: Participants suggested having a central, organized repository for all the resources	• PLC facilitators were more intentional about when and how resources were shared. PLC members were shown where the resources were located and how to access the shared Google Workspace

## Discussion

The findings from this developmental evaluation demonstrate the role that a PLC can play in supporting teachers to create not only LGBTQIA+-inclusive classroom content, but also on creating more inclusive school environments through increased skill building among staff. Findings showed how the PLC was structured yet flexible enough to allow for the development of teachers’ own projects. Those projects were aligned with understanding students’ identities at an individual level, developing inclusive curricula at a classroom level, and leading policy implementation at a school level. Findings also revealed that participants reported value in terms of learning and community-building. A number of these key findings relate to the extant literature in a variety of ways and help us to understand the value of this PLC. These are described below.

As noted above, one of the theories grounding this work is the Social-Ecological Model (SEM) as this is how the district's strategy to support LGBTQIA+ students is framed across multiple levels (e.g. supporting students through policies at the district level, through GSAs at the school/organizational level, and through required PD at the individual level). As described in the paper, the PLC allowed teacher participants to develop and innovate their own projects aligned not only with the goal of creating inclusive curricula, but more broadly with the district's vision of supporting LGBTQIA+ students. As described in Table 1, teacher participants developed a variety of projects that coincidentally aligned with the SEM. The curriculum projects aim to foster student understanding and learning at the individual level. The student survey project aims to foster improved understanding and affirm students’ identities at the interpersonal level. Finally, the professional development project aims to expand upon already required PD to foster discussion and implement practices to support LGBTQIA+ students at the organizational, or school, level. Together these projects can function synergistically to foster multi-level change. This aligns with what we know is needed for systems and culture change of this nature (Golden & Earp, 2012).

While many approaches may have been effective in supporting teacher professional development (PD), the PLC model was chosen for its ability to provide teachers with a safe space to explore and take risks outside of their local school context where they may feel some level of fear or anxiety about making mistakes (Kelly, 2017; Stoll et al., 2006; Van Lare & Brazer, 2013). Trust and respect among members are an important characteristic of a successful PLC (Grimm, 2024; Kelly, 2017; Stoll et al., 2006; Van Lare & Brazer, 2013). The development and adherence to group norms and values, as well as the focus on community building activities, promoted trust and respect among GI PLC members, which fostered a safe space for members. PLCs also allowed for a responsive and flexible scope and sequence where the learning sequence could be adjusted in response to participant feedback. Teacher feedback directly led to the expansion of approaches beyond curriculum projects to include student-centered teaching through survey design and school culture and climate support via professional development for staff. This PLC was intentionally organized at the district level to allow teachers to build community with other invested practitioners to deepen their understanding, collaborate on projects, and receive critical feedback. Positioning the PLC at the district level also created the possibility of amplifying and sharing the projects and learning more widely throughout the district.

This evaluation only begins to address a gap in curricula when it comes to LGBTQIA+-inclusivity. Illinois is one of a small number of states in which LGBTQIA+ inclusive curricula are legally required. Most of these laws specifically state that social studies and/or sexual health education be inclusive of LGBTQIA+ identities (citation: Inclusive Curricular Standards Policies | GLSEN: Navigator). However, for science curricula, there is no such mandate or guidance. That said, best practices in this area are emerging at the national level (Calaway, 2020; Cooper & Berry, 2020; Wright & Delgado, 2023). There are general frameworks for guiding principles (Wright & Delgado, 2023) as well as specific science frameworks and curricular models (Long et al., 2021; Rende Mendoza & Johnson, 2024). In this project, the gender-inclusive biology website served as a wellspring of information, guidance, and ideas (Long, 2019). There is a need to extend LGBTQIA+ inclusive practices into other nontraditional courses as well, such as math and other STEM topics. Despite the growing availability of these models and curricula, more work is needed to introduce these techniques to teachers, encourage their utilization, and support them in integrating them into their existing curricula. This study demonstrates that a PLC approach is appropriate for continuing to contribute to the creation and implementation of inclusive science curricula.

Further, there is a need to support educators who identify as transgender in leading this work. As noted in the literature, educators who identify as cisgender and/or heterosexual who try to be inclusive may need additional support and guidance from transgender educators who have led the way and can ensure attempts at inclusivity do not fall short (Rende Mendoza & Johnson, 2024). This PLC had a facilitator who is transgender as well as two TA providers who identified as transgender and/or nonbinary. There is an opportunity to further develop this work with a focus on LGBTQIA+ educators, and transgender educators in particular.

This was a single PLC in a state and district with policies supportive of LGBTQIA+ young people and therefore the findings are not generalizable. However, there remain a few key recommendations that may be transferable to other similarly situated districts. First, whereas some content and skill-building related to LGBTQIA+-inclusivity works well to be included in a structured mandatory training, such as ensuring policies protecting the rights of students to use names, pronouns, and facilities that correspond with their identity, other dimensions of LGBTQIA+-inclusivity better-suited for other forms of PD. Curriculum development, for which there are fewer examples, and for which creativity and teacher ownership are required, a PLC is a powerful vehicle to support teachers’ own development and innovation of projects. Secondly, the facilitation of such a PLC must be iterative and flexible to allow for creativity and for teachers to create what they perceive as most relevant and needed for their schools and classrooms. Allowing teacher agency and flexibility in learning and projects aligns with what we know to be key characteristics of adult learning theory and of high-quality PLCs, although in practice we know PLCs are not always facilitated in such a way. Adhering to this PLC principle is essential when working in such an exploratory area. Finally, a focus on curriculum should not come at the exclusion of a focus on the classroom and school environment or a focus on relationship-building with students. We know that LGBTQIA+ students are most protected when their schools adhere to protective policies (Kosciw et al., 2020, 2022) and when they have positive relationships with trusted adults in school (Truong et al., 2021; Zongrone et al., 2022). Being able to formulate such strategies together, rather than in a siloed manner, likely has greater potential to be impactful on the lives of LGBTQIA+ students.

## Limitations

Like all studies, this one is not without limitations. First, this small case study may be subject to participation bias. Of the 9 participants in the PLC, only 5 chose to participate in an interview. While those who participated reported uniformly positive experiences and provided constructive feedback with a stated desire to improve the PLC and continue to participate, it is possible that those who chose not to participate had different experiences that are therefore not captured here. Secondly, participants were not asked to disclose their own sexual identity or their gender identity upon participation in the PLC, nor upon participation in the evaluation interview. While it is imperative to center LGBTQIA+ identities in this work, the authors believe it is equally important to be inclusive of all identities to ensure that all staff have the capacity to support all students. That said, the impossibility of describing our sample and better contextualizing this inaugural PLC is a missed opportunity.

## Conclusion

While CPS had engaged in efforts to educate teachers and school staff and ensure curricula were inclusive in sexual health education and social studies, there remained an opportunity to engage in more participatory PD approaches and to ensure LGBTQIA+-inclusivity in science curricula. The CPS Gender Inclusivity PLC aimed to create a space for teachers to engage in critical reflection and to begin to develop science curricula. The PLC proved fruitful ground to create multi-level strategies that can work in tandem to better support students on an interpersonal level, within the science classroom, and within their school communities. The CPS Gender Inclusivity PLC can be a model for other curricular teams in CPS as well as for other districts working to support LGBTQIA+ students and engage in multi-level change approaches.
